# Evaluation of Quality Changes in Huajiao Seed Oil During Different Storage Conditions

**DOI:** 10.3390/foods15101708

**Published:** 2026-05-13

**Authors:** Xiaowei Peng, Bofei Fu, Haibo Liu, Cuilan Fang, Jianquan Kan

**Affiliations:** 1 College of Food Science, Southwest University, Chongqing 400715, China; qqajx19@163.com (X.P.); fubofeit@163.com (B.F.); 2 Jiulongpo Center for Disease Control and Prevention, Chongqing 400039, China; 3 College of Food Science and Engineering, Xinyang Agriculture and Forestry University, Xinyang 464000, China; luke9502@163.com

**Keywords:** Huajiao seed oil, quality, storage conditions, fatty acids, volatile composition

## Abstract

Huajiao seed oil is a high-quality edible vegetable oil that is rich in unsaturated fatty acids. Because of this characteristic, it exhibits poor stability and is prone to oxidation. However, storage methods significantly influence oxidative stability. Therefore, this study investigated the effects of temperature (4, 25, and 37 °C), light exposure, and packaging materials (glass bottle, PET bottle, and iron can) on the quality of Huajiao seed oil during storage. The results demonstrate that low temperature effectively retarded the increase in acid value, peroxide value, *p*-anisidine value, and the content of secondary oxidation products. It also slowed down the degradation of squalene and *α*-tocopherols. Prolonged light exposure accelerated the oxidative rancidity of Huajiao seed oil. Oil stored in glass bottles exhibited a lower degree of oxidation than that stored in PET bottles or iron cans, and when stored under conditions of 4 °C/glass bottle/darkness, it had a shelf life of up to 7.34 months. The main volatile compounds generated in Huajiao seed oil during storage were aldehydes and acids.

## 1. Introduction

Huajiao seed oil, extracted from the seeds of *Zanthoxylum bungeanum*, is a distinctive plant oil that has garnered significant attention owing to its unique flavor profile, high nutritional value, and potential bioactive compounds [1]. As an essential component in the comprehensive utilization of Huajiao by-products, this oil is particularly rich in unsaturated fatty acids such as linoleic and α-linolenic acid, as well as vitamin E, sterols, and polyphenols [2]. Consequently, it holds considerable promise for applications in the food, pharmaceutical, and cosmetic industries. The chemical composition of Huajiao seed oil dictates its high oxidation sensitivity. Research indicates that the unsaturated fatty acid content can exceed 80%, with polyunsaturated fatty acids constituting over 50% [3,4]. Although elevated polyunsaturated fatty acid levels confer antioxidant and anti-inflammatory properties, they also render the oil highly susceptible to auto-oxidation and enzymatic oxidation under conditions such as light exposure, elevated temperatures, and oxygen contact. This leads to the formation of harmful by-products, including peroxides and aldehydes [5]. Moreover, the characteristic flavor compounds and natural antioxidants in the oil may degrade or interact during storage, further accelerating lipid deterioration. These changes not only compromise the sensory quality of the oil but may also introduce potential health risks [6].

Extensive research has been conducted on the influence of environmental factors (temperature, light, oxygen, humidity) and packaging materials on the quality of vegetable oil. Studies have shown that the oxidative stability of sunflower and olive oils is significantly negatively correlated with storage temperature [7,8]. Low temperatures (4–20 °C) effectively inhibit free radical chain reactions, while exposure to light, particularly ultraviolet light, accelerates photo-oxidation, leading to increased peroxide values and conjugated diene content [9]. Packaging materials also impact the oils’ oxidation rate [10]. However, studies have focused on common vegetable oils such as soybean and rapeseed oils, whereas systematic research on specialty oils such as Huajiao seed oil remains limited. Furthermore, due to the unique lipid composition of Huajiao seed oil, its storage stability may differ from that of common edible oils [11]. This is mainly reflected in changes in basic physicochemical indicators, functional components, and oxidation products. Understanding these patterns of change has important implications for the utilization of Huajiao seed oil.

Huajiao seed oil is susceptible to oxidative rancidity, color deterioration, and the degradation of functional components during storage owing to environmental factors. These changes significantly reduce its nutritional quality, sensory characteristics, and commercial value. However, these quality deteriorations are not only dependent on the type of oil but also closely related to the storage conditions. Consequently, this study, for the first time, systematically investigated the effects of temperature (4, 25, 37 °C), light exposure (darkness, lighting), packaging material (glass bottle, polyethylene terephthalate (PET) bottle, iron can), and storage duration (0, 2, 4, 6, 8, 10, 12 months) on the physicochemical properties, fatty acid composition, and volatile flavor compounds of Huajiao seed oil. Research findings elucidate the relationship between the oxidative stability of Huajiao seed oil and storage parameters, which holds significant implications for optimizing storage protocols, extending shelf life, and enhancing the utilization value of Huajiao seed oil resources.

## 2. Materials and Methods

### 2.1. Materials and Reagents

Huajiao seed oil was provided by Jiaofangdian Agricultural Science and Technology Co. (Longnan, China). The glass bottles (250 mL), PET bottles (250 mL), and iron cans (250 mL) were all food-grade and purchased from Ningjin Hengtong Metal Products Co., Ltd. (Dezhou, China). Squalene and α-tocopherol were sourced from Yuanye Bio-Technology Co., Ltd. (Shanghai, China). Propanal, (E)-2-pentenal, (E)-2-hexenal, n-hexanal, (E)-2-nonenal, and (E,E)2,4-decadienal were purchased from Aladdin Bio-Chem Technology Co. Ltd. (Shanghai, China).

### 2.2. Design Storage Conditions

Huajiao seed oil was aliquoted into glass bottles, PET bottles, and iron cans, with each container filled with 200 mL of the oil sample. Container caps were secured tightly during storage. To simulate typical household storage practices for edible oils, Huajiao seed oil was stored under the conditions listed in Table 1. To mitigate positional bias, samples were gently agitated at 7-day intervals, and their positions within incubators or refrigerators were randomly redistributed. The total storage period was 12 months, with quality evaluations performed bimonthly. All three container types had a total capacity of 250 mL. Ten distinct storage protocols were implemented, with three independently stored oil samples under each protocol, resulting in a total of 30 experimental samples.

### 2.3. Determination of Basic Physicochemical Parameters

The acid value was determined according to GB 5009.229-2016 [12], the peroxide value according to GB 5009.227-2023 [13], the anisidine value according to GB/T 24304-2024 [14], the 2-thiobarbituric acid value according to GB/T 35252-2017 [15], and the carbonyl value according to GB 5009.230-2016 [16]. The color (L*, a*, and b*) was measured using a colorimeter (CM-5, Konica Minolta, Tokyo, Japan).

### 2.4. Establishment of Oxidation Kinetic Model

The oxidation process of Huajiao seed oil under different storage conditions was modeled using a first-order oxidation kinetic model, and the shelf life was predicted with peroxide value (0.25 g/100 g) as the evaluation indicator [17,18]. The first-order oxidation kinetic equation is as follows:

(1) $$C=C_{0}\times e^{\left(kt\right)}$$

where *C* is the peroxide value, *k* is the rate constant, *C*_0_ is the initial peroxide value, and *t* is the storage time.

### 2.5. Determination of Trace Composition

#### 2.5.1. Squalene

The squalene, β-sitosterol, stigmasterol and campesterol in the Huajiao seed oil were determined by gas chromatography (GC, 7890A, Agilent, Santa Clara, CA, USA). Briefly, 0.5 g of Huajiao seed oil was weighed and placed in a 25 mL tube; then, 1 mL of 1 mol/L KOH–methanol solution was added, and the mixture was vortexed for 2 min. Subsequently, 5 mL of n-hexane was added, and the mixture was vortexed for 1 min. The mixture was washed with saturated sodium chloride solution until neutral. Then 3 mL of the upper phase was transferred to a 10 mL tube, 0.3 g of anhydrous sodium sulfate was added for drying, and the solution was filtered through a 0.22 μm membrane [19]. GC conditions: DB-5MS capillary column (30.0 m × 0.35 mm × 0.25 μm) with an FID, injection volume 1.0 μL, nitrogen flow rate 1.0 mL/min, injector temperature 250 °C, and detector temperature 300 °C. The initial column temperature was set to 160 °C, held for 1 min, then increased to 220 °C at a rate of 15 °C/min, and held for 2 min. Subsequently, the temperature was further increased to 280 °C at a rate of 5 °C/min, held for 10 min, and then increased to 300 °C at a rate of 10 °C/min, which was maintained for 2 min. Squalene was quantified using the external standard method.

#### 2.5.2. *α*-Tocopherol

Determination of α-tocopherol in Huajiao seed oil was conducted according to Li et al.’s method [20]. Specifically, 2.0 g of Huajiao seed oil was dissolved in 5.0 mL of anhydrous ethanol and subjected to ultrasonic extraction for 15 min. Following centrifugation, the supernatant was collected, filtered through a 0.22 μm membrane, and analyzed using high-performance liquid chromatography (HPLC, 1260, Agilent, USA). The HPLC conditions were as follows: a C18 column (4.6 mm × 250 mm, 5 μm, Agilent, USA) was employed, the detection wavelength was set to 294 nm, the mobile phase consisted of methanol, the flow rate was maintained at 1.0 mL/min, the column temperature was kept at 20 °C, and the injection volume was 5.0 μL. Qualitative and quantitative analysis of *α*-tocopherol in the Huajiao seed oil was performed based on the retention time and standard curve.

### 2.6. Analysis of Fatty Acid Composition

Saponification was performed by adding 250 mg of Huajiao seed oil (0, 6, 12 months) to 8 mL of KOH–methanol solution (2%, *w*/*w*) at 80 °C. Subsequently, 7 mL of boron trifluoride methanol solution (14%, *w*/*w*) was added, and the mixture was heated for an additional 2 min. After cooling to room temperature, 20 mL of n-hexane was added, and the mixture was vortexed for 2 min. Saturated sodium chloride solution was then added, and the mixture was allowed to stand until phase separation occurred. The upper layer (5 mL) was transferred to a 25 mL test tube, and approximately 4 g of anhydrous sodium sulfate was added. The mixture was vortexed for 1 min and left to stand for 5 min. The upper layer was then filtered through a 0.22 μm membrane for GC analysis [21].

GC analysis: A gas chromatograph equipped with a capillary column of polydimethylsiloxane strong polar stationary phase (100 m × 0.25 mm, 0.25 μm, Supelco, Bellefonte, PA, USA) and a flame ionization detector (FID) were used to analyze fatty acids. The injection volume was 1.0 μL, detector temperature 280 °C, nitrogen flow rate 1.0 mL/min, and injector temperature 270 °C. The initial column temperature was set to 100 °C, held for 13 min, then increased at a rate of 10 °C/min to 180 °C and held for 6 min, increased at 1 °C/min to 200 °C, held for 20 min, and finally increased at 4 °C/min to 230 °C and held for 10.5 min. Fatty acid types in Huajiao seed oil were identified by comparing the relative retention times of sample peaks with those of fatty acid methyl ester standards; results were expressed as the percentage of each fatty acid relative to the total fatty acids.

### 2.7. Analysis of Secondary Oxidation Products

Determination of secondary oxidation products in Huajiao seed oil (0, 6, 12 months) was performed according to Cao et al.’s method [22], slightly modified. Briefly, 0.3 g of oil sample or secondary oxidation product standard was dissolved in 1.0 mL of 3.0 g/L 2,4-dinitrophenylhydrazine ethanol solution and reacted at 40 °C for 1 h. After centrifugation at 4200 rpm for 5 min, 20 μL of the supernatant was filtered through a 0.22 μm membrane and analyzed by HPLC (1260, Agilent, USA). The HPLC conditions were as follows: the mobile phase was a mixture of acetonitrile and water (75:25, *v*/*v*) with a flow rate of 1.0 mL/min, the detection wavelength was 365 nm, and the column temperature was maintained at 40 °C.

### 2.8. Analysis of Volatile Composition

#### 2.8.1. Extraction of Volatile Components

Volatile components in Huajiao seed oil (12 months) were extracted by headspace solid-phase microextraction. Briefly, 2.0 g of the oil was placed in a 20 mL headspace vial, with 20 μL of 2-octanol (0.8445 μg/L) added as internal standard. The mixture was balanced at 60 °C and 300 r/min for 15 min, and then the solid-phase microextraction needle (50/30 µm, DVB/CAR/PDMS; Supelco, USA) was inserted and adsorbed for 30 min under the same conditions [23].

#### 2.8.2. Identification of Volatile Components

The extraction head was withdrawn from the headspace vial and immediately inserted into the GC injection port for thermal desorption at 250 °C for 5 min. A quartz capillary column (DB-5MS, 30 m × 0.25 mm × 0.25 μm) was used. The temperature program was: hold at 60 °C for 10 min, increase at 2 °C/min to 70 °C, then increase at 10 °C/min to 140 °C, followed by an increase at 5 °C/min to 170 °C with a 1 min hold, and finally increase at 15 °C/min to 230 °C with a 4 min hold. The injection port temperature was set to 250 °C. Helium (He, purity 99.999%) was used as the carrier gas with a flow rate of 0.8 mL/min and a pressure of 37.8 kPa. The injection volume was 1 μL. The electron impact ionization source had an electron energy of 70 eV, with an ion source temperature of 230 °C, interface temperature of 230 °C, and mass scanning range of 40–400 m/z.

All data obtained from GC-MS analysis were processed by automatic deconvolution using AMDIS (version 2.72) and qualitatively identified using the NIST23.L mass spectral library. The processed data were exported in Excel format and imported into SIMCA 14.1 software to establish an orthogonal partial least squares discriminant analysis (OPLS-DA) model. Subsequently, a 7-fold cross-validation and 200 permutation tests were performed. The variable importance in projection (VIP) was used to evaluate the contribution of each variable in the OPLS-DA model. Variables with VIP > 1.0 and *p* < 0.05 were considered differential compounds.

### 2.9. Statistical Analysis

All experiments were conducted in triplicate using independently prepared samples, and results are presented as the mean ± standard deviation. Homogeneity of variance was verified prior to ANOVA, and no violations were observed. While the sample size is modest, it is appropriate for the controlled laboratory conditions employed and aligns with common practices in oil-related research. Data visualization was performed using Origin 2018, and statistical analysis of differences was conducted using SPSS Statistics 26. Statistical differences were analyzed using one-way analysis of variance, and *p* < 0.05 was defined as statistically significant.

## 3. Results

### 3.1. Change in Color of Huajiao Seed Oil During Storage

**Figure 1:**
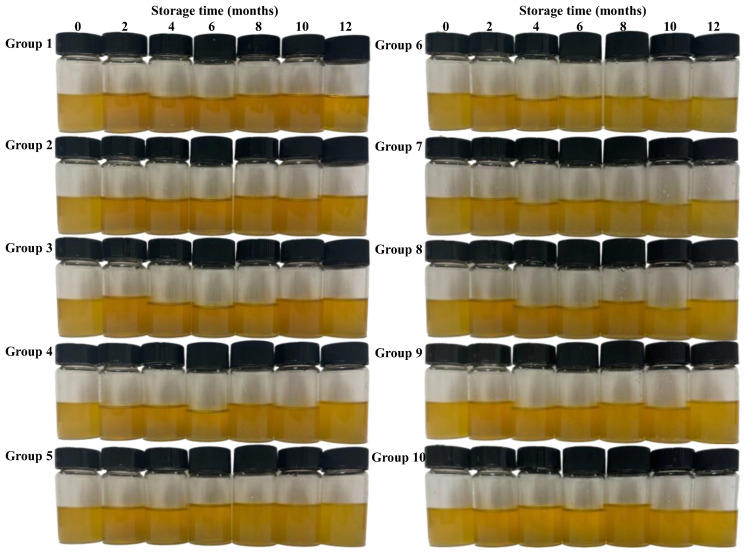
Appearance of Huajiao seed oil during storage.

**Figure 2:**
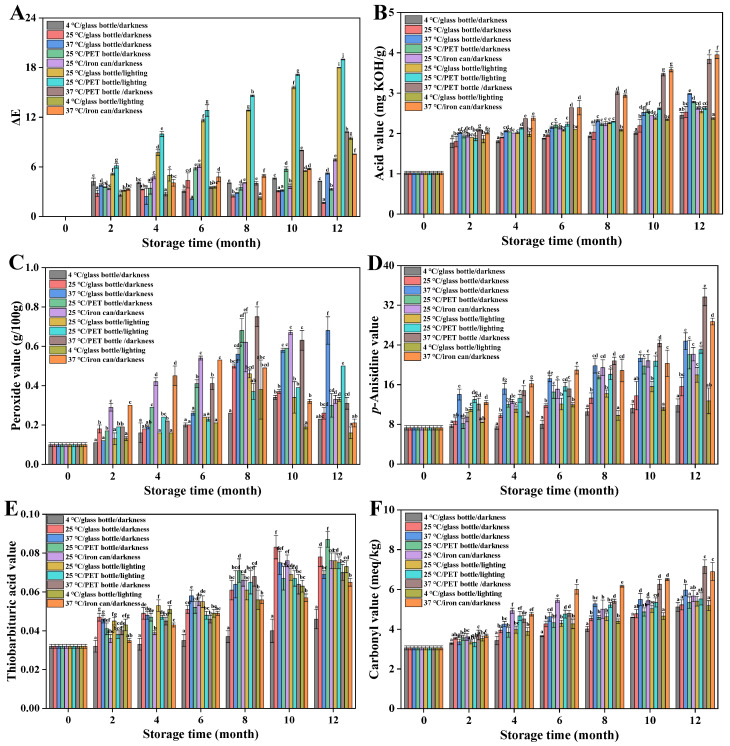
Changes in basic physicochemical parameters of Huajiao seed oil during 12 months of storage. (**A**) Δ*E*; (**B**) Acid value; (**C**) Oxidation value; (**D**) *p*-Anisidine value; (**E**) Thiobarbituric acid value; (**F**) Carbonyl value. Different letters (a–j) indicate significant differences (*p* < 0.05) between groups under different storage conditions at the same stage.

Color is the most visually apparent characteristic of oils and is directly linked to type, freshness, and nutritional quality [24]. Appearance of Huajiao seed oil under different storage conditions are shown in Figure 1. Analysis showed that fresh Huajiao seed oil exhibited the following color parameters: lightness (L*) = 86.43, red–green value (a*) = −1.36, and yellow–blue value (b*) = 60.17. The color of all oil groups changed to varying degrees in different storage conditions. As detailed in Table S1, lightness (L*) of all Huajiao seed oil groups generally increased during storage. Among them, groups 1, 2, and 4 exhibited the smallest degree of change, whereas groups 5, 8, and 10 demonstrated the largest degree of change. The a* value decreased in almost all groups (Table S2). The reduction was relatively minor in groups 1 and 4, whereas group 8 exhibited the largest decrease. This suggests that the color of Huajiao seed oil gradually shifts from red to green during storage. The b* values changed under different storage conditions. The b* values of groups 1, 2, and 3 showed a slight increase during storage, whereas those of the remaining groups significantly decreased and were negatively correlated with storage duration (Table S3). Groups 6 and 7 experienced the largest decrease, suggesting that the yellow hue of Huajiao seed oil in these groups faded most prominently. Comparison of the color difference (Δ*E*) values among the groups is presented in Figure 2A. While controlling the packaging material, elevated temperatures and light exposure accelerated the alteration in oil color, with light exerting a far greater impact than temperature. Under controlled temperature and light conditions, glass bottles preserved color better than PET and iron cans. Previous studies indicated that pigment components in edible oils are broken down during storage, gradually lightening the color [25]. However, research also confirmed that new pigment components may be formed in edible oils during storage, resulting in a deeper color [26]. During the 12-month storage period, the oil’s color did not change unidirectionally, indicating concurrent generation and degradation of soluble dyestuffs within the oil.

### 3.2. Changes in Acid Value and Peroxide Value of Huajiao Seed Oil During Storage

The acid value, an indicator of oils’ free fatty acid content, results from the hydrolysis of glycerides catalyzed by microorganisms, enzymes, heat, moisture, and metal ions, and it is widely employed as a key quality parameter for oils [27]. Acid values of all oil groups exhibited varying degrees of increase during storage (Figure 2B). A relatively rapid increase in acid value occurred during the initial storage phase (0–2 months), rising from an initial value of 1.02 mg KOH/g to 1.76–2.08 mg KOH/g. Temperature exerted the most significant influence on the acid value of the oil. After 12 months’ storage, acid value ranges were 2.37–2.45 mg KOH/g at 4 °C, 2.53–2.78 mg KOH/g at 25 °C, and 2.98–3.95 mg KOH/g at 37 °C. Exposure to light increased the oil’s acid value. Under controlled temperature and packaging material conditions, the acid values of light-exposed Huajiao seed oil samples were consistently higher than those of samples kept in darkness (Figure 2B). Packaging material exerted a discernible effect on the acid value. Under identical storage conditions, the oil stored in glass bottles consistently exhibited lower acid values than samples stored in PET bottles or iron cans. Therefore, heat is an important factor that increases Huajiao seed oil’s acid value, and among the packaging materials evaluated, glass bottles proved the most suitable packaging, outperforming PET bottles and iron cans.

During the initial stage of oil oxidation, unsaturated fatty acids are activated by light, heat, and metal catalysts. This activation facilitates the abstraction of a hydrogen atom from the methylene carbon adjacent to the double bond, forming an unstable alkyl radical (R·). In the presence of oxygen, this radical combines with molecular oxygen to generate a peroxyl radical (ROO·). The content of these peroxyl radicals is quantified by the peroxide value [28]. The peroxide values of all Huajiao seed oil groups exhibited varying degrees of increase during 0–8 months’ storage (Figure 2C). The peroxide values of oil stored in iron cans demonstrated a significantly higher rate of increase than the other groups, possibly attributable to the catalytic effect of metal ions. Elevated temperatures did not consistently increase the peroxide values. Under controlled packaging materials and identical light conditions, oils stored at 37 °C showed lower peroxide values than those at 4 °C and 25 °C. This observation does not indicate a lower degree of oxidation in the high-temperature-treated oil; it is highly probable that peroxides undergo premature cleavage at elevated temperatures, generating secondary oxidation products, resulting in reduced peroxide levels [29]. In most of the oil groups under study, peroxide values gradually decreased after 8–12 months’ storage. This occurs when peroxides reach saturation within the system and subsequently undergo rapid decomposition into secondary oxidation products, including aldehydes, ketones, and acids [30]. Therefore, to comprehensively evaluate the degree of oxidation of Huajiao seed oil during storage, it is essential to analyze secondary oxidation products formed during the oxidation process.

### 3.3. Change in p-Anisidine Value of Huajiao Seed Oil During Storage

Peroxides generated during oil oxidation are unstable and decompose to form aldehydes. Among these, *α*,*β*-unsaturated aldehydes react with *p*-anisidine under acidic conditions to form a yellow chromophore [31]. The content of *α*,*β*-unsaturated aldehydes in the oil can be quantified by measuring the reaction product. The p-anisidine values for all oil groups under study increased progressively with extended storage time (Figure 2D). Oils stored at elevated temperature (37 °C) exhibited a significantly faster rate of *p*-anisidine value increase, reaching values of 24.77–33.67 after 12 months’ storage. Conversely, oils stored at low temperature (4 °C) had substantially lower p-anisidine values (11.79–12.73) after 12 months. Compared to groups stored in darkness, samples exposed to light consistently demonstrated higher p-anisidine values, irrespective of storage temperature (4, 25, or 37 °C). Under otherwise identical storage conditions, Huajiao seed oil stored in glass bottles consistently exhibited lower p-anisidine values than oils stored in PET bottles or iron cans.

### 3.4. Change in Thiobarbituric Acid Value of Huajiao Seed Oil During Storage

Malondialdehyde is a characteristic oxidation product formed from unsaturated fatty acids, particularly polyunsaturated fatty acids that contain three or more double bonds, such as linolenic acid [32]. Thiobarbituric acid values of the different groups increased progressively during storage (Figure 2E). Oil stored under 4 °C/glass bottle/light conditions exhibited the slowest increase in thiobarbituric acid value, reaching only 0.046 after 12 months’ storage. By contrast, all other groups underwent significantly faster increases, indicating that both light and heat promote the formation of malondialdehyde. Among the other nine storage conditions, thiobarbituric acid values showed almost no obvious differences at the same storage time points.

### 3.5. Change in Carbonyl Value of Huajiao Seed Oil During Storage

Carbonyl compounds, primarily aldehydes and ketones, generated from the oxidation of unsaturated fatty acids in oils, are quantified by their carbonyl value. This parameter is a critical indicator for assessing the extent of oxidative deterioration in oils [33]. The oil’s carbonyl value increased to varying degrees during storage (Figure 2F). Samples stored at elevated temperature (37 °C) exhibited significantly higher carbonyl values than other groups after 12 months’ storage, reaching values of 5.97–7.15 meq/kg. In contrast, low-temperature groups, particularly those stored at 4 °C, demonstrated a slower rate of carbonyl value increase. Light exposure promoted the formation of carbonyl compounds, whereas storage in glass bottles retarded it. These observations are consistent with the conclusions drawn earlier.

### 3.6. Oxidation Kinetic Model

The fitted curves for changes in the peroxide value of Huajiao seed oil during the 12-month storage period are shown in Table 2. The *R*^2^ values of all fitting equations ranged from 0.8472 to 0.9951, indicating a certain degree of fit and a reasonable ability to describe the oxidation extent of Huajiao seed oil. Furthermore, using a peroxide value of 0.25 g/100 g as the critical point, the shelf life of Huajiao seed oil under different storage conditions was predicted. It was found that the samples stored under conditions of 4 °C/glass bottle/darkness and 4 °C/glass bottle/lighting had longer shelf lives of 7.34 and 6.08 months, respectively. In contrast, those stored under conditions of 25 °C/iron can/darkness and 37 °C/iron can/darkness exhibited shorter shelf lives of only 1.48 and 1.71 months, respectively. It can be seen that under the iron can/darkness conditions, the predicted shelf life of the 25 °C group is 0.23 months shorter than that of the 37 °C group. The reason for this result is not that the degree of oxidation at 37 °C is lower than that at 25 °C, but rather a possible fitting deviation of the first-order kinetic equation.

### 3.7. Change in Trace Composition of Huajiao Seed Oil During Storage

**Figure 3:**
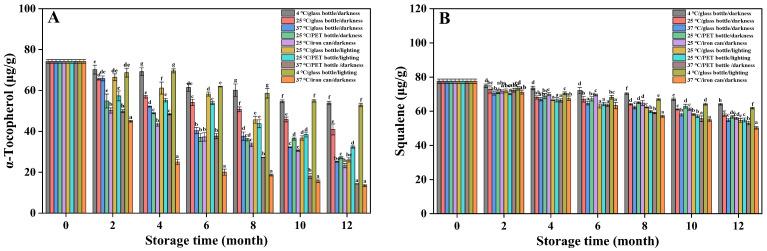
Changes in the content of α-tocopherol and squalene in Huajiao seed oil during 12 months of storage. (**A**) α-Tocopherol; (**B**) Squalene. Different letters (a–h) indicate significant differences (*p* < 0.05) between groups under different storage conditions at the same stage.

α-Tocopherol is a significant minor component in vegetable oils, conferring certain sensory and functional properties. It inhibits deterioration and extends edible oils’ shelf lives [34]. The α-tocopherol content decreased after 12 months’ storage under different conditions (Figure 3A). Storage at 4 °C resulted in the least degradation of α-tocopherol, with minimal influence from light exposure. After 12 months, the α-tocopherol contents in groups 1 and 9 were 53.84 μg/g and 52.89 μg/g, respectively. However, significantly greater losses occurred at 37 °C, with groups 3, 8, and 10 declining to 25.19 μg/g, 14.31 μg/g, and 13.46 μg/g after 12 months’ storage. Analysis of groups 2, 4, and 5 showed that, under otherwise identical conditions, Huajiao seed oil stored in glass bottles exhibited significantly higher α-tocopherol retention compared to oil stored in PET bottles or iron cans.

Squalene imparts nutritional value to vegetable oils, such as antioxidant and anti-aging properties, making it a significant indicator for assessing edible oil quality. However, owing to the presence of six unsaturated bonds in its structure, squalene is highly unstable and readily oxidized [35]. The squalene content under different storage conditions is presented in Figure 3B. The squalene content in fresh Huajiao seed oil was 77.54 μg/g, gradually decreasing during storage. Storage conditions significantly impacted squalene content. Low temperatures favored squalene retention; after 12 months’ storage, the squalene content in groups 1 and 9 was significantly higher than in other groups. In the high-temperature groups (groups 3, 8, and 10), squalene content was consistently lower than in the low-temperature groups throughout the storage period. Comparative analysis of groups 1 and 9, 2 and 6, and 4 and 7 showed that light exposure led to a reduction in squalene content, all other conditions being constant. Finally, the squalene content in oil packaged in glass bottles was higher than that in PET bottles and iron cans.

### 3.8. Changes in Secondary Oxidation Products of Huajiao Seed Oil During Storage

**Figure 4:**
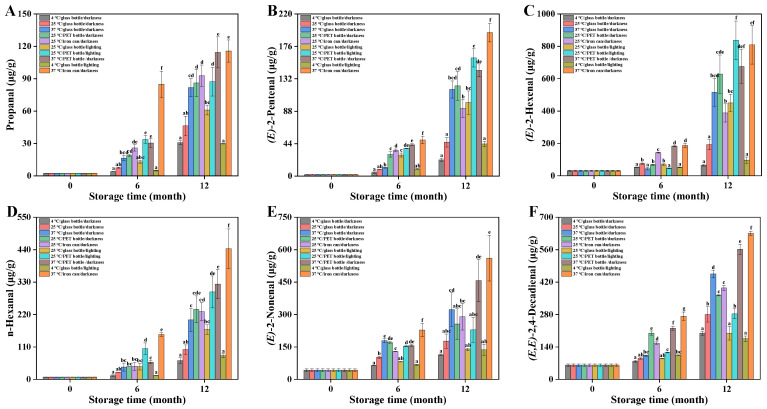
Changes in secondary oxidation products in Huajiao seed oil under different storage conditions. (**A**) Propanal (**B**) (E)-2-Pentenal; (**C**) (E)-2-Hexenal; (**D**) n-hexanal; (**E**) (E)-2-nonenal; (**F**) (E,E)-2,4-decadienal. Different letters (a–g) indicate significant differences (*p* < 0.05) between groups under different storage conditions at the same stage.

During the initial stages of lipid oxidation, unsaturated fatty acids react with oxygen to form unstable hydroperoxides. As oxidation progresses, these hydroperoxides accumulate and, upon reaching a certain concentration, decompose into secondary oxidation products, such as aldehydes, ketones, and acids [29]. Parameters such as p-anisidine, thiobarbituric acid, and carbonyl values provide a general indication of secondary oxidation products in oils. However, the specific identification and quantification of individual secondary oxidation products require chromatographic analysis. The changes in six aldehyde secondary oxidation products during storage are presented in Figure 4. Prior to storage, the contents of propanal, (E)-2-pentenal, (E)-2-hexenal, n-hexanal, (E)-2-nonenal, and (E,E)2,4-decadienal in the oil were 2.45 μg/g, 1.98 μg/g, 30.83 μg/g, 7.51 μg/g, 42.59 μg/g, and 61.11 μg/g, respectively. Fresh Huajiao seed oil contained low levels of aldehydes, probably generated during the oil refining process. After 6 and 12 months’ storage under various conditions, the content of all six aldehydes increased significantly. This increase was particularly pronounced between months 6 and 12. Storage conditions significantly affected the aldehyde content in the oil. The increase in aldehydes was relatively slow in group 1. After 12 months’ storage, the propanal, (E)-2-pentenal, (E)-2-hexenal, n-hexanal, (E)-2-nonenal, and (E,E)2,4-decadienal contents reached 30.74 μg/g, 21.99 μg/g, 64.16 μg/g, 63.73 μg/g, 113.77 μg/g, and 199.21 μg/g, respectively. Aldehyde accumulation was likewise slow in group 9 because the low temperature retarded oil oxidation. The high-temperature groups (3, 8, and 10) exhibited rapid increases in aldehyde compounds. However, even when stored at the same temperature (37 °C), the aldehyde contents in groups 3, 8, and 10 showed significant differences. The contents of all six aldehydes were significantly lower in oil stored in glass bottles compared to PET bottles or iron cans, indicating that glass packaging retarded the oxidation process. Light exposure promoted the formation of aldehydes in the oil, but its effect was minimal at 4 °C. The six aldehydes showed almost no significant differences between groups 1 and 9. However, when stored at 25 °C, the contents of (E)-2-pentenal and (E)-2-hexenal were significantly higher in light-exposed oil than in light-protected oil.

Secondary oxidation products are primarily generated by the oxidation of unsaturated fatty acids. Specific oxidation products vary according to the fatty acid type. Studies indicate that n-hexanal, (E,E)-2,4-decadienal, and (E)-2-nonenal predominantly originate from the oxidative decomposition of linoleic acid [9,22]. Therefore, it is imperative to analyze the fatty acid composition of Huajiao seed oil under various storage conditions.

### 3.9. Changes in Fatty Acid Composition of Huajiao Seed Oil During Storage

**Figure 5:**
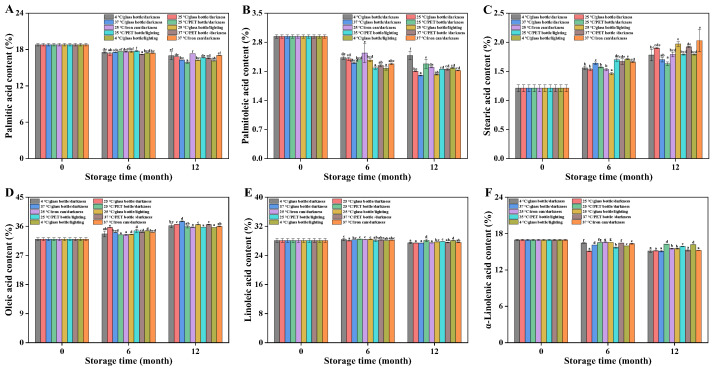
Changes in fatty acid content under different storage conditions. (**A**) Palmitic acid (**B**) Palmitoleic acid; (**C**) Stearic acid; (**D**) Oleic acid; (**E**) Linoleic acid; (**F**) α-Linolenic acid. Different letters (a–g) indicate significant differences (*p* < 0.05) between groups under different storage conditions at the same stage.

Fatty acids serve as critical substrates in lipid oxidation processes by reacting with oxygen and generating aldehydes and ketones under catalysis by light and metal ions, thereby inducing oil quality deterioration. Previous studies identified that Huajiao seed oil predominantly comprises palmitic acid (18.79%), palmitoleic acid (2.94%), stearic acid (1.21%), oleic acid (32.02%), linoleic acid (28.08%), and α-linolenic acid (16.96%), with polyunsaturated fatty acids constituting approximately 45% of the total profile, indicating high oxidative susceptibility [36]. Storage under varying conditions for 6 months resulted in decreased levels of palmitic acid, palmitoleic acid, and α-linolenic acid alongside increased stearic and oleic acid contents, while linoleic acid showed no significant change (Figure 5). After 12 months’ storage, palmitic acid, palmitoleic acid, and *α*-linolenic acid exhibited continued reduction; stearic and oleic acids demonstrated further elevation; and linoleic acid displayed a declining trend. The consumption of linoleic acid and particularly α-linolenic acid—attributable to oxidation driven by their multiple unsaturated double bonds—explains the observed relative increases in oleic and stearic acids, which stem from the diminished proportions of palmitic acid, palmitoleic acid, linoleic acid, and α-linolenic acid [37].

Storage conditions significantly impacted the oil’s fatty acid composition. The *α*-linolenic acid content in group 3 declined to 15.10% after 12 months’ storage, significantly lower than those of fresh oil (16.96%) and all other storage groups, indicating the most pronounced oxidation of α-linolenic acid under these conditions (Figure 5F). Conversely, group 4 exhibited the slowest rate of α-linolenic acid reduction, decreasing from 16.96% to 16.26%. Storage under group 1 conditions also resulted in a decrease in α-linolenic acid (16.96% to 15.11%), demonstrating measurable oxidation even at low temperature in the absence of light. Beyond α-linolenic acid, linoleic acid was also readily oxidized. Changes in linoleic acid content across storage conditions are shown in Figure 5E; most groups exhibited varying degrees of reduction over 0–12 months. Mirroring the *α*-linolenic acid results, group 3 showed a significant decline in linoleic acid after 12 months (28.08% to 27.41%), while groups 4 and 9 displayed no significant change. At the 6-month mark, most groups showed no reduction in linoleic acid compared to fresh oil, likely due to its lower oxidation priority compared to α-linolenic acid, consistent with Wang et al.’s findings with rapeseed oil [38]. The highest oxidation level for palmitoleic acid was observed in group 3, decreasing from 2.94% to 2.00%. Collectively, these results demonstrate that polyunsaturated fatty acids in Huajiao seed oil, particularly α-linolenic acid, are oxidatively degraded during storage, leading to reduced content.

### 3.10. Changes in Volatile Composition of Huajiao Seed Oil During Storage

**Figure 6:**
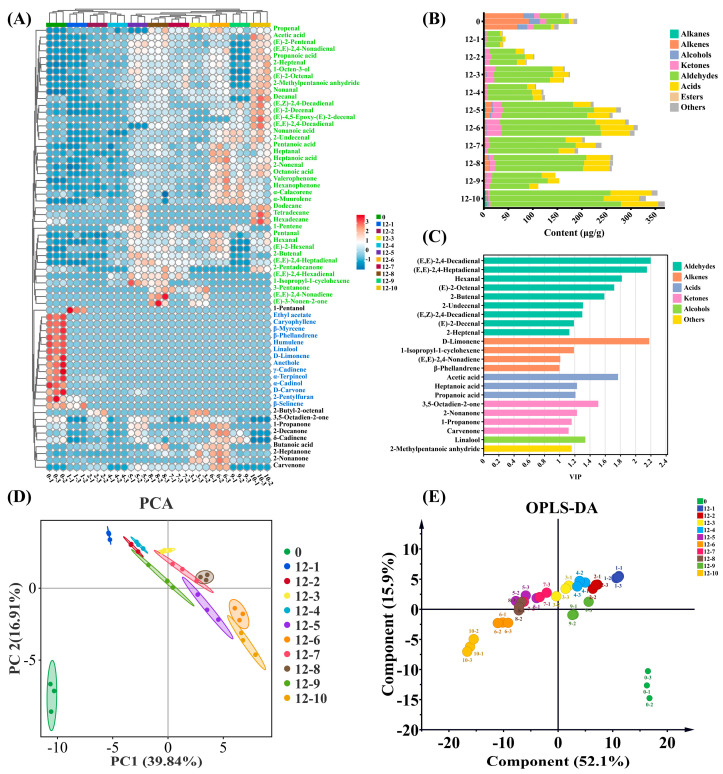
Changes in volatile composition under different storage conditions. Cluster heatmap analysis (**A**), content of various compounds (**B**), VIP values (**C**), PCA score plot (**D**), OPLS-DA score plot (**E**).

Storage not only leads to loss of inherent flavor compounds present in Huajiao seed oil but also causes oxidative rancidity, generating unpleasant odors. These odor compounds primarily consist of the oil’s oxidation products, such as aldehydes, ketones, and acids [39]. However, storage conditions significantly affect both the flavor profile and the degree of oxidation. As shown in Table S4, 65 volatile compounds were identified, including 13 alkenes, 12 ketones, 21 aldehydes, 7 acids, 5 alcohols, 3 alkanes, 1 ester, and 3 others. Changes in the types and concentrations of volatile substances under each storage condition are illustrated in Figure 6B. The volatile compound content in fresh oil was 172.29 μg/g, primarily consisting of alkenes, aldehydes, alcohols, and ketones, with concentrations of 79.47 μg/g, 41.90 μg/g, 21.92 μg/g, and 12.50 μg/g, respectively. After 12 months’ storage, significant changes occurred in the oil’s volatile composition. Group 10 exhibited the highest increase in total volatile content, reaching 351.70 μg/g, followed by groups 6, 8, and 5, in which it increased to 306.08 μg/g, 263.14 μg/g, and 252.22 μg/g, respectively. Regarding compound classes, this increase was largely attributed to the generation of substantial amounts of oil oxidation products (aldehydes, acids, ketones) [40]. Conversely, the concentrations of alkenes and alcohols decreased markedly after 12 months’ storage, representing the primary cause of flavor loss in the oil. The total volatile compound content in group 1 was only 41.10 μg/g, significantly lower than that of fresh oil. This group failed to generate large quantities of volatile oxidation products such as aldehydes, acids, and ketones, and the original alkenes and alcohols present in the oil were nearly completely depleted. This indicates that while these conditions (4 °C/glass bottle/darkness) can effectively delay oil oxidation and prevent the formation of large amounts of oxidation products, they are insufficient for preserving the oil’s original flavor characteristics.

Cluster heatmap analysis was performed on the 65 volatile compounds (Figure 6A). The volatile composition of the oil exhibited distinct differences under various storage conditions. Ethyl acetate, caryophyllene, *β*-myrcene, *β*-phellandrene, 2-pentylfuran, and *β*-selinene (blue markers) were the predominant volatile components in fresh Huajiao seed oil. However, their contents were almost entirely lost through volatilization after storage. Propenal, acetic acid, (E)-2-pentenal, (E,E)-2,4-nonadienal, (E,E)-2,4-nonadiene, and (E)-3-nonen-2-one (green markers) were volatile compounds that were newly formed or significantly increased in most groups (except for groups 1, 2, and 4) after 12 months’ storage. Notably, 1-pentanol was found at relatively high levels only in group 1.

To further analyze the impact of storage conditions on the oil’s volatile composition, principal component analysis (PCA) was used to examine differences among the groups [41]. Fresh Huajiao seed oil was located in the third quadrant of the PCA score plot, whereas the stored oils were located in the first, second, and fourth quadrants (Figure 6D), indicating a significant alteration in the volatile composition of the oil after 12 months’ storage. The oils from groups 1, 2, and 4 clustered within the second quadrant, suggesting similarities in their volatile profiles. This similarity may be associated with their storage under non-elevated temperatures (4 and 25 °C) and light-protected conditions. Conversely, the oils from groups 5 and 10 were positioned in the fourth quadrant, implying the influence of the iron packaging material. Compared to the unsupervised nature of PCA, supervised orthogonal projections to latent structure discriminant analysis (OPLS-DA) offers the advantage of minimizing within-group variation while maximizing between-group differences, resulting in superior separation [42]. Distinct clustering trends were evident among groups (Figure 6E). The model parameters (*R_x_*^2^ = 0.981, *R_y_*^2^ = 0.903, *Q*^2^ = 0.764) indicate a well-fitted OPLS-DA model. Validation using a 200-cycle permutation test (Figure S1) yielded intercepts of *R*^2^ = 0.36 and *Q*^2^ = −0.856 with the *y*-axis, confirming the absence of overfitting. Based on the criteria of VIP > 1 and *p* < 0.05, 22 key volatile compound markers were screened (Figure 6C). These included (E,E)-2,4-decadienal, D-limonene, (E,E)-2,4-heptadienal, hexanal, acetic acid, (E)-2-octenal, 2-butenal, 3,5-octadien-2-one, and linalool.

In summary, while low temperatures can delay the oxidation of Huajiao seed oil and reduce the generation of volatile oxidation products such as aldehydes, ketones, and acids, they cannot prevent the loss of inherent flavor compounds caused by volatilization during prolonged storage. Light exposure promotes oxidation, leading to the formation of compounds such as aldehydes and acids, even at low temperatures. The packaging material significantly influences the volatile composition of the oil. Under light-protected conditions at either 25 or 37 °C, the levels of aldehydes and acids in oil packaged in glass bottles were consistently lower than those in PET bottles or iron cans.

## 4. Discussion

The stability of Huajiao seed oil during storage is significantly impacted by environmental factors such as temperature, light exposure, packaging materials, and storage duration. Temperature is the primary driver of oxidative rancidity in Huajiao seed oil. Low-temperature preservation (4 °C) effectively retarded lipid oxidation, as evidenced by minimal changes in the peroxide value and acid value after 12 months [43]. Elevated storage temperature (37 °C) accelerated primary and secondary oxidation, leading to higher peroxide values in oils stored under high temperature during the initial stage of oxidation. However, as oxidation progresses, the peroxide values of oils stored at lower temperatures may exceed those of oils stored at higher temperatures. This is primarily because elevated temperatures also promote the degradation of primary oxidation products, resulting in a decrease or a slower increase in the peroxide value [11]. The changes in the peroxide value of Huajiao seed oil at different temperatures observed in this study support this view. In addition, elevated temperatures also lead to rapid degradation of α-tocopherol and pronounced increases in the acid value and *p*-anisidine value. This correlates with accelerated radical chain propagation and hydrolytic rancidity [44]. Oils stored at room temperature (25 °C) exhibited intermediate degradation rates, indicating that refrigeration is required for long-term preservation of the oil. The barrier properties of packaging materials are crucial for preserving oil quality during storage. Our results indicate that glass bottles provided superior preservation efficacy for Huajiao seed oil, retaining over 70% of the initial α-tocopherol after 12 months at 4 °C. Their impermeability to oxygen and ultraviolet light effectively hindered photo-oxidation and auto-oxidation. PET bottles permitted gradual oxygen ingress, leading to higher peroxide values than glass after 12 months [38]. Their partial ultraviolet transparency exacerbated the degradation of α-tocopherols and squalene and the generation of oxidation products under light exposure. Although iron cans completely blocked light, they exhibited unexpected oxidation acceleration. This is attributable to trace metal ions (Fe^2+^) leached from the can interior catalyzing Fenton-type reactions [45]. The effects of photo-oxidation on the storage of edible oil cannot be overlooked. Light exposure significantly amplified α-tocopherol degradation and volatile aldehyde formation at 25 °C. This is attributed to ultraviolet-triggered homolytic cleavage of hydroperoxides [46]. During storage, Huajiao seed oil experiences significant loss of original flavor compounds such as D-limonene, linalool, β-myrcene, and caryophyllene, with noticeable volatilization occurring even at 4 °C. Consequently, future research should explore natural antioxidants or preservation methods for Huajiao seed oil that address the dual objectives of inhibiting lipid oxidation and preserving the oil’s characteristic flavor composition.

## 5. Conclusions

Storage conditions significantly affected the quality of Huajiao seed oil. Lower temperature (4 °C) more effectively retards the increase in oxidation indices, such as the acid value, peroxide value, p-anisidine value, and content of secondary oxidation products. Lower temperatures also better preserve the content of squalene and α-tocopherol, thereby extending the oil’s shelf life. Light exposure accelerates the oxidative rancidity of the oil and promotes the accumulation of oxidation products. Oil stored in glass bottles exhibits lower oxidation indices than oil stored in PET bottles or iron cans. Linoleic and α-linolenic acids are the primary substrates oxidized during the storage of Huajiao seed oil. After 12 months’ storage, although low temperature reduced the levels of volatile compounds such as aldehydes and acids, it did not prevent the inevitable loss of the oil’s original flavor components. Low temperature combined with light protection effectively delays the oxidative rancidity of Huajiao seed oil, and glass bottles demonstrated superior performance compared to other packaging materials. Therefore, without considering economic factors, glass packaging with low light transmittance has potential for the storage of Huajiao seed oil. This study provides valuable practical guidance for selecting storage methods for Huajiao seed oil.

## Figures and Tables

**Table 1:** Design of the storage experiment on Huajiao seed oil.

Groups	Temperature	Packaging Material	Light Conditions	Storage Time
1	4 °C	Glass bottle	Darkness	12 months
2	25 °C	Glass bottle	Darkness	12 months
3	37 °C	Glass bottle	Darkness	12 months
4	25 °C	PET bottle	Darkness	12 months
5	25 °C	Iron can	Darkness	12 months
6	25 °C	Glass bottle	Lighting	12 months
7	25 °C	PET bottle	Lighting	12 months
8	37 °C	PET bottle	Darkness	12 months
9	4 °C	Glass bottle	Lighting	12 months
10	37 °C	Iron can	Darkness	12 months

Note: PET bottles and glass bottles were both made of colorless and transparent materials. If the experiment was conducted under dark conditions, they were wrapped with aluminum foil. Under light conditions, natural light was used.

**Table 2:** Kinetic model of oxidation of Huajiao seed oil under different conditions.

Groups	Storage Conditions	Parameters		*R* ^2^	RMSE	Shelf Life (Month)
		*C*_0_	*k*			
1	4 °C/glass bottle/darkness	0.0924	0.1299	0.9947	0.0055	7.34 ± 0.38
2	25 °C/glass bottle/darkness	0.0755	0.2256	0.8472	0.0541	5.31 ± 1.27
3	37 °C/glass bottle/darkness	0.1267	0.1469	0.9519	0.0764	4.63 ± 1.13
4	25 °C/PET bottle/darkness	0.1077	0.2296	0.9951	0.0124	3.64 ± 0.50
5	25 °C/iron can/darkness	0.2002	0.1499	0.8959	0.0596	1.48 ± 0.79
6	25 °C/glass bottle/lighting	0.0689	0.2321	0.9471	0.0258	5.56 ± 0.89
7	25 °C/PET bottle/lighting	0.1388	0.1074	0.9384	0.0332	5.28 ± 1.07
8	37 °C/PET bottle/darkness	0.0853	0.2698	0.9819	0.0269	3.99 ± 0.94
9	4 °C/glass bottle/lighting	0.0787	0.1903	0.9316	0.0204	6.08 ± 1.12
10	37 °C/iron can/darkness	0.1806	0.1900	0.8782	0.0571	1.71 ± 0.69

## Data Availability

The original contributions presented in this study are included in the article/Supplementary Materials. Further inquiries can be directed to the corresponding authors.

## Supplementary Materials

The following supporting information can be downloaded at https://www.mdpi.com/article/10.3390/foods15101708/s1: Figure S1: Results of 200 permutation tests; Figure S2: Chromatogram of fatty acids; Table S1: Changes in lightness (*L**) of Huajiao seed oil during 12-month storage; Table S2: Changes in red–green value (*a**) of Huajiao seed oil during 12-month storage; Table S3: Changes in yellow–blue value (*b**) of Huajiao seed oil during 12-month storage; Table S4: Changes in the types and contents (μg/g) of volatile components in huajiao seed oil after 12 months of storage under different conditions.
