# Supplementary material for: Evaluation of Quality Changes in Huajiao Seed Oil During Different Storage Conditions
Source: Foods. 2026 May 13;15(10):1708. doi: 10.3390/foods15101708 (PMC13206043; doi:10.3390/foods15101708)
Supplement: Supplementary file 1 [file foods-15-01708-s001.zip › foods-4217710-supplementary.pdf]

Supplementary Materials

Table S1. Changes in lightness (L^*) of Huajiao seed oil during 12-month storage.

Groups	Storage conditions	0 months	2 months	4 months	6 months	8 months	10 months	12 months
1	4 ℃/glass bottle/darkness	86.43±0.11	87.26±0.53^{a}	88.09±0.16^{a}	88.75±0.13^{a}	87.14±0.19^{a}	86.42±0.12^{a}	89.55±0.02^{c}
2	25 ℃/glass bottle/darkness	86.43±0.11	87.87±0.20^{b}	88.74±0.18^{bc}	89.62±0.73^{bc}	87.40±0.42^{ab}	87.97±0.02^{c}	87.34±0.24^{a}
3	37 ℃/glass bottle/darkness	86.43±0.11	88.10±0.12^{b}	88.14±0.22^{ab}	88.49±1.16^{a}	87.37±0.44^{ab}	88.70±0.06^{d}	91.11±0.07^{e}
4	25 ℃/PET bottle/darkness	86.43±0.11	89.75±0.06^{ef}	90.15±1.03^{d}	90.03±0.15^{bc}	88.98±0.31^{de}	87.63±0.30^{b}	88.72±0.17^{b}
5	25 ℃/iron can/darkness	86.43±0.11	89.90±0.11^{fg}	90.97±0.32^{e}	91.46±0.34^{d}	87.86±0.09^{c}	89.29±0.24^{e}	92.10±0.11^{g}
6	25 ℃/glass bottle/lighting	86.43±0.11	90.16±0.14^{g}	90.66±0.44^{de}	91.39±0.19^{d}	89.53±0.05^{f}	90.17±0.19^{f}	91.37±0.16^{f}
7	25 ℃/PET bottle/lighting	86.43±0.11	90.64±0.14^{h}	90.37±0.16^{de}	91.27±0.34^{d}	90.38±0.23^{g}	90.41±0.11^{f}	91.97±0.04^{g}
8	37 ℃/PET bottle /darkness	86.43±0.11	88.91±0.17^{c}	89.27±0.21^{c}	89.47±0.47^{b}	88.75±0.16^{d}	91.87±0.03^{h}	92.95±0.05^{i}
9	4 ℃/glass bottle/lighting	86.43±0.11	89.45±0.11^{d}	90.54±0.06^{de}	89.77±0.03^{bc}	87.77±0.16^{bc}	88.87±0.11^{d}	90.89±0.13^{d}
10	37 ℃/iron can/darkness	86.43±0.11	89.55±0.15^{de}	88.82±0.54^{c}	90.37±0.17^{c}	89.28±0.22^{ef}	90.97±0.08^{g}	92.39±0.04^{h}

Note: different letters (a-i) indicate significant differences ($p<0.05$) between groups under different storage conditions at the same stage.

Table S2. Changes in red–green value (a^*) of Huajiao seed oil during 12-month storage.

Groups	Storage conditions	0 months	2 months	4 months	6 months	8 months	10 months	12 months
1	4 °C/glass bottle/darkness	-1.36±0.01	-0.38 ± 0.04^{h}	-0.72 ± 0.13^{e}	-1.51 ± 0.26^{d}	-0.26 ± 0.08^{f}	0.17 ± 0.07^{i}	-1.76 ± 0.01^{f}
2	25 °C/glass bottle/darkness	-1.36±0.01	-1.07 ± 0.04^{g}	-1.50 ± 0.25^{d}	-2.34 ± 0.31^{c}	-0.91 ± 0.15^{e}	-0.88 ± 0.05^{h}	-0.93 ± 0.09^{g}
3	37 °C/glass bottle/darkness	-1.36±0.01	-1.03 ± 0.06^{g}	-1.39 ± 0.14^{d}	-1.86 ± 0.38^{d}	-0.93 ± 0.23^{e}	-1.97 ± 0.02^{f}	-3.38 ± 0.05^{d}
4	25 °C/PET bottle/darkness	-1.36±0.01	-2.02 ± 0.05^{e}	-2.70 ± 0.55^{b}	-2.58 ± 0.59^{bc}	-2.42 ± 0.33^{c}	-1.71 ± 0.08^{g}	-2.20 ± 0.03^{e}
5	25 °C/iron can/darkness	-1.36±0.01	-2.40 ± 0.03^{d}	-3.44 ± 0.16^{a}	-3.79 ± 0.13^{a}	-2.41 ± 0.03^{c}	-2.86 ± 0.13^{d}	-3.89 ± 0.05^{c}
6	25 °C/glass bottle/lighting	-1.36±0.01	-2.73 ± 0.11^{b}	-3.50 ± 0.08^{a}	-3.68 ± 0.05^{a}	-2.70 ± 0.04^{b}	-3.09 ± 0.10^{c}	-3.37 ± 0.05^{d}
7	25 °C/PET bottle/lighting	-1.36±0.01	-3.31 ± 0.04^{a}	-3.43 ± 0.03^{a}	-3.77 ± 0.10^{a}	-3.03 ± 0.09^{a}	-2.80 ± 0.06^{d}	-3.36 ± 0.03^{d}
8	37 °C/PET bottle /darkness	-1.36±0.01	-1.88 ± 0.11^{f}	-2.42 ± 0.03^{bc}	-2.84 ± 0.08^{b}	-2.41 ± 0.10^{c}	-4.67 ± 0.02^{a}	-5.42 ± 0.03^{a}
9	4 °C/glass bottle/lighting	-1.36±0.01	-1.97 ± 0.06^{ef}	-3.52 ± 0.17^{a}	-2.71 ± 0.04^{bc}	-1.45 ± 0.09^{d}	-2.38 ± 0.06^{e}	-3.39 ± 0.07^{d}
10	37 °C/iron can/darkness	-1.36±0.01	-2.55 ± 0.14^{c}	-2.10 ± 0.24^{c}	-3.46 ± 0.12^{a}	-3.09 ± 0.08^{a}	-3.90 ± 0.05^{b}	-4.70 ± 0.01^{b}

Note: different letters (a-i) indicate significant differences ($p<0.05$) between groups under different storage conditions at the same stage.

Table S3. Changes in yellow–blue value (b^*) of Huajiao seed oil during 12-month storage.

Groups	Storage conditions	0 months	2 months	4 months	6 months	8 months	10 months	12 months
1	4 °C/glass bottle/darkness	60.17±0.07	64.19±0.33^{i}	63.85±0.14^{f}	62.14±0.40^{d}	63.99±0.01^{i}	64.49±0.05^{i}	63.03±0.04^{j}
2	25 °C/glass bottle/darkness	60.17±0.07	62.57±0.33^{g}	62.47±0.14^{e}	58.13±2.85^{c}	62.38±0.02^{g}	62.78±0.10^{h}	61.45±0.05^{i}
3	37 °C/glass bottle/darkness	60.17±0.07	63.57±0.16^{h}	61.72±1.27^{e}	60.83±.041^{d}	62.80±0.06^{h}	62.24±0.08^{g}	61.13±0.02^{h}
4	25 °C/PET bottle/darkness	60.17±0.07	61.53±0.20^{f}	59.35±0.18^{d}	55.35±0.23^{b}	58.03±0.05^{e}	54.59±0.33^{c}	57.97±0.04^{g}
5	25 °C/iron can/darkness	60.17±0.07	59.70±0.05^{d}	56.78±0.24^{c}	54.98±0.13^{b}	56.51±0.01^{c}	58.47±0.08^{f}	57.30±0.03^{f}
6	25 °C/glass bottle/lighting	60.17±0.07	56.32±0.11^{b}	52.99±0.55^{b}	49.10±0.13^{a}	47.80±0.04^{b}	45.12±0.11^{b}	42.98±0.04^{b}
7	25 °C/PET bottle/lighting	60.17±0.07	54.94±0.26^{a}	50.54±0.27^{a}	47.72±0.66^{a}	46.22±0.03^{a}	43.57±0.06^{a}	42.11±0.03^{a}
8	37 °C/PET bottle /darkness	60.17±0.07	61.02±0.04^{e}	59.48±0.06^{d}	57.72±0.35^{c}	57.08±0.10^{d}	55.37±0.01^{d}	53.44±0.01^{d}
9	4 °C/glass bottle/lighting	60.17±0.07	61.08±0.07^{e}	55.97±1.03^{c}	58.19±0.23^{c}	58.40±0.03^{f}	55.36±0.02^{d}	52.08±0.09^{c}
10	37 °C/iron can/darkness	60.17±0.07	58.51±0.14^{c}	57.29±2.39^{c}	56.09±0.71^{b}	56.56±0.02^{c}	57.71±0.03^{e}	57.01±0.02^{e}

Note: different letters (a-j) indicate significant differences ($p<0.05$) between groups under different storage conditions at the same stage.

Table S4. Changes in the types and contents (μg/g) of volatile components in huajiao seed oil after 12 months of storage under different conditions.

Compounds	Formula	CAS	Classification	0	12-1	12-2	12-3	12-4	12-5	12-6	12-7	12-8	12-9	12-10	*p* value	VIP
Propenal	C_3H_4O	107-02-8	Aldehydes	-	0.85±0.12	0.86±0.17	1.52±0.80	0.64±0.18	-	3.27±1.06	2.57±0.10	3.32±1.01	2.02±0.81	2.64±0.35	5.66E-07	0.87
Acetic acid	$C_2H_4O_2$	64-19-7	Acids	-	1.24±0.33	5.64±1.29	7.78±2.79	9.32±0.24	19.91±4.04	17.52±2.16	16.00±1.47	25.23±3.51	4.68±0.88	35.76±7.03	5.05E-12	1.77
3-Pentanone	$C_5H_{10}O$	96-22-0	Ketones	-	-	-	0.20±0.07	-	-	-	0.13±0.09	0.18±0.07	-	-	1.04E-06	0.25
Ethyl acetate	$C_4H_8O_2$	141-78-6	Esters	2.16±0.06	-	-	-	-	-	-	-	-	-	-	6.31E-24	0.44
2-Butenal	C_4H_6O	4170-30-3	Aldehydes	0.16±0.03	1.59±0.27	-	7.38±1.16	6.36±0.81	18.17±1.86	12.68±1.22	11.20±1.75	11.71±2.06	1.37±0.44	3.49±1.28	1.31E-14	1.59
Propanoic acid	$C_3H_6O_2$	79-09-4	Acids	-	0.52±0.02	3.29±0.51	6.51±1.65	3.15±0.62	8.77±0.25	10.29±0.76	5.83±0.41	9.74±0.27	-	14.44±0.35	1.74E-18	1.21
Pentanal	$C_5H_{10}O$	110-62-3	Aldehydes	1.22±0.20	1.37±0.66	0.89±0.28	3.60±0.44	1.82±0.32	4.55±0.54	4.86±1.30	3.21±0.81	3.08±0.35	1.68±0.35	3.56±1.01	3.74E-07	0.87
(E)-2-Pentenal	C_5H_8O	1576-87-0	Aldehydes	-	0.69±0.14	1.34±0.39	1.63±0.44	2.29±0.39	5.92±0.68	3.29±0.15	3.40±0.44	5.55±0.76	-	6.31±0.28	8.85E-16	0.86
1-Pentanol	$C_5H_{12}O$	71-41-0	Alcohols	-	0.72±0.15	-	-	-	-	-		-	-	-	2.47E-14	0.58
1-Pentene	C_5H_{10}	109-67-1	Alkenes	-	-	-	-	0.67±0.14	1.00±0.25	-	0.89±0.27	-	0.73±0.23	-	2.98E-10	0.62
Butanoic acid	$C_4H_8O_2$	107-92-6	Acids	-	0.22±0.02	0.41±0.01	0.91±0.42	-	-	1.44±0.17	-	1.25±0.33	-	-	6.81E-11	0.64
Hexanal	$C_6H_{12}O$	66-25-1	Aldehydes	6.37±1.38	3.25±0.52	6.94±1.34	14.95±4.13	14.88±0.61	24.75±2.44	32.36±5.09	24.69±3.34	19.40±1.71	5.13±1.42	25.76±1.38	2.11E-11	1.82
(E)-2-Hexenal	$C_6H_{10}O$	6728-26-3	Aldehydes	-	0.37±0.02	0.26±0.04	0.44±0.05	0.34±0.10	0.96±0.25	1.33±0.21	0.60±0.17	0.98±0.19	-	0.78±0.07	7.90E-11	0.45
Pentanoic acid	$C_5H_{10}O_2$	109-52-4	Acids	2.45±0.26	1.92±0.53	3.36±0.24	5.46±1.54	2.70±1.43	5.34±1.12	8.90±3.34	4.35±1.08	6.97±1.16	6.49±2.42	8.52±1.50	8.16E-05	0.91
2-Heptanone	$C_7H_{14}O$	110-43-0	Ketones	0.33±0.11	0.42±0.07	0.33±0.03	1.95±0.88	0.38±0.01	0.69±0.05	2.82±0.26	0.93±0.19	1.10±0.39	0.70±0.19	0.71±0.09	2.08E-08	0.73
Heptanal	$C_7H_{14}O$	111-71-7	Aldehydes	0.55±0.05	0.53±0.14	0.44±00.3	0.73±0.11	0.50±0.07	1.27±0.13	2.25±0.10	0.99±0.23	1.35±0.14	1.12±0.26	1.28±0.45	1.94E-09	0.44
(E,E)-2,4-Hexadienal	C_6H_8O	142-83-6	Aldehydes	-	-	-	-	0.30±0.18	0.74±0.20	-	0.54±0.23	0.78±0.17	-	-	2.83E-09	0.45
1-Isopropyl-1-cyclohexene	C_9H_{16}	4292-04-0	Alkenes	1.32±0.50	0.51±0.11	-	0.67±0.19	1.25±0.40	6.32±1.54	-	-	4.38±0.48	-	-	1.50E-12	1.19

(E,E)-2,4-Nonadiene	C_9H_{16}	56700-78-8	Alkenes	-	-	-	1.54±0.90	-	-	-	-	4.47±1.04	-	-	5.65E-11	1.01
2-Heptenal	$C_7H_{12}O$	18829-55-5	Aldehydes	1.18±0.45	2.44±0.27	5.69±0.52	7.57±1.90	4.32±0.34	10.78±1.45	11.17±1.35	8.84±2.43	8.83±0.55	3.24±0.36	13.96±2.89	1.59E-09	1.13
β-Phellandrene	$C_{10}H_{16}$	555-10-2	Alkenes	11.19±1.07	-	-	-	-	-	-		-	-	-	1.48E-21	1.00
1-Octen-3-ol	$C_8H_{16}O$	3391-86-4	Alcohols	-	0.42±0.08	0.77±0.07	1.10±0.36	0.83±0.16	2.26±0.32	1.67±0.20	1.31±0.33	1.76±0.17		2.75±0.43	5.44E-12	0.53
β-Myrcene	$C_{10}H_{16}$	123-35-3	Alkenes	9.08±0.89	-	-	-	-	-	-	--		-	-	2.82E-21	0.90
2-Pentylfuran	$C_9H_{14}O$	3777-69-3	Others	3.36±1.09	1.11±0.33	-	-	-		--	-	-	-	-	3.95E-10	0.65
(E,E)-2,4-Heptadienal	$C_7H_{10}O$	4313-03-5	Aldehydes	7.30±0.48	4.62±0.33	11.33±0.67	27.18±6.84	20.96±5.75	36.39±11.92	24.67±1.73	32.54±1.52	38.31±7.69	7.49±0.94	26.41±1.32	4.29E-08	2.15
D-Limonene	$C_{10}H_{16}$	5989-27-5	Alkenes	53.11±9.19	0.56±0.12	0.16±0.03	-	-	1.03±0.19	-	--		-	-	6.14E-16	2.18
(E)-2-Octenal	$C_8H_{14}O$	2548-87-0	Aldehydes	-	0.95±0.42	5.74±1.14	10.62±0.47	8.38±2.24	17.68±4.16	18.07±3.26	18.58±5.57	12.56±1.07	-	28.36±2.43	1.52E-11	1.72
2-Methylpentanoic anhydride	$C_{12}H_{22}O_3$	63169-61-9	Others	-	-	1.82±0.57	2.62±0.40	4.03±1.71	7.38±1.86	8.37±0.81	8.00±3.36	5.32±0.75	-	12.96±1.12	2.53E-10	1.16
3,5-Octadien-2-one	$C_8H_{12}O$	38284-27-4	Ketones	7.39±0.36	0.45±0.02	5.30±1.93	7.43±1.40	1.95±0.77	7.44±1.21	6.11±0.38	1.09±0.22	4.41±1.22	2.68±1.17	-	7.97E-10	1.51
Heptanoic acid	$C_7H_{14}O_2$	111-14-8	Acids	2.09±0.71	0.67±0.12	2.91±0.81	3.54±0.19	1.59±0.76	4.92±0.31	15.21±1.47	5.33±1.32	4.95±0.27	7.07±1.69	10.01±1.32	2.61E-13	1.23
2-Nonanone	$C_9H_{18}O$	821-55-6	Ketones	-	0.39±0.15	-	3.76±0.66	-	-	4.76±0.86	-	-	0.95±0.40	-	2.25E-14	1.23
Linalool	$C_{10}H_{18}O$	78-70-6	Alcohols	20.40±0.79	0.12±0.01	-		--	0.90±0.03	-	-	-	0.87±0.19	-	9.48E-30	1.34
Nonanal	$C_9H_{18}O$	124-19-6	Aldehydes	-	1.64±0.64	3.46±0.56	5.63±0.62	2.34±0.49	4.36±0.29	6.97±0.44	4.94±1.64	6.98±0.64	3.83±0.19	10.68±0.98	8.77E-13	0.94
(E)-3-Nonen-2-one	$C_9H_{16}O$	18402-83-0	Ketones	-	-	-	0.24±0.04	-	-	-	-	0.45±0.16	-	-	3.36E-10	0.32
2-Nonenal	$C_9H_{16}O$	18829-56-6	Aldehydes	2.98±0.62	1.96±0.92	3.87±0.92	4.90±0.81	3.67±0.44	7.24±1.37	10.01±1.26	5.09±1.32	6.35±0.45	7.15±1.26	10.63±0.81	1.28E-09	0.92
1-Propanone	$C_9H_{10}O$	93-55-0	Ketones	2.00±0.47	-	1.81±0.56	-	--	3.85±0.38	5.44±0.50	-		-	-	4.01E-17	1.16
Octanoic acid	$C_8H_{16}O_2$	124-07-2	Acids	1.41±0.43	0.42±0.10	1.50±0.48	2.58±0.34	1.37±0.23	3.22±0.77	9.28±1.78	4.41±0.42	4.07±0.64	5.19±1.74	8.75±1.08	1.01E-10	0.93
α-Terpineol	$C_{10}H_{18}O$	10482-56-1	Alcohols	1.10±0.14	0.12±0.06	0.22±0.04	-	-	-	-	-	--	-	-	1.32E-17	0.33

2-Decanone	$C_{10}H_{20}O$	693-54-9	Ketones	1.27±0.49	0.34±0.04	1.14±0.32	1.09±0.22	0.42±0.11	2.71±0.64	4.42±0.64	1.37±0.57	1.32±0.51	1.87±0.71	-	4.24E-09	0.83
Dodecane	$C_{12}H_{26}$	112-40-3	Alkanes	-	0.16±0.04	0.27±0.03	-	-	1.71±0.21	-	--	-	-	1.97±0.18	1.29E-19	0.72
Decanal	$C_{10}H_{20}O$	112-31-2	Aldehydes	0.46±0.10	-	0.29±0.07	0.94±0.04	0.23±0.04	0.51±0.07	0.94±0.10	0.47±0.11	1.01±0.05	-	2.10±0.30	2.61E-15	0.52
(E,E)-2,4-Nonadienal	$C_9H_{14}O$	5910-87-2	Aldehydes	-	-	1.30±0.61	2.58±0.21	2.12±0.26	6.37±0.83	6.49±1.66	3.21±0.61	5.58±0.71	-	7.83±1.57	1.42E-11	0.97
D-Carvone	$C_{10}H_{14}O$	2244-16-8	Ketones	0.86±0.19	-	-	-	-	0.18±0.08	-	-	-	-	-	4.26E-13	0.28
Carvenone	$C_{10}H_{16}O$	499-74-1	Ketones	-	0.25±0.02	0.61±0.03	0.73±0.12	-	0.95±0.21	1.72±0.09	0.71±0.17	0.78±0.08	-	-	1.13E-15	1.12
(E)-2-Decenal	$C_{10}H_{18}O$	3913-81-3	Aldehydes	3.97±0.61	1.58±0.61	3.40±0.88	4.69±0.16	4.41±0.92	10.27±1.54	12.29±1.44	9.09±1.75	9.23±0.50	10.50±2.82	17.55±2.88	2.91E-10	0.64
Nonanoic acid	$C_9H_{18}O_2$	112-05-0	Acids	1.66±0.43	0.42±0.12	1.12±0.46	1.80±0.34	-	2.89±0.70	5.39±0.93	3.65±1.35	3.02±0.36	5.76±2.08	6.51±1.15	4.28E-08	0.97
Anethole	$C_{10}H_{12}O$	104-46-1	Others	4.31±0.75	-	-	-	-	-		--	-	-	-	6.66E-16	0.62
(E,Z)-2,4-Decadienal	$C_{10}H_{16}O$	25152-83-4	Aldehydes	2.54±0.54	1.26±0.39	2.05±0.73	3.67±0.27	2.52±0.52	8.48±1.11	6.57±0.89	3.67±0.21	8.43±0.47	9.07±3.89	14.39±3.41	2.47E-08	1.30
(E,E)-2,4-Decadienal	$C_{10}H_{16}O$	25152-84-5	Aldehydes	9.09±1.94	4.24±0.44	8.21±2.16	13.90±3.77	9.34±1.60	-	26.04±4.48	14.28±1.24	29.39±1.44	26.13±3.11	48.68±11.45	3.82E-11	2.20
Valerophenone	$C_{11}H_{14}O$	1009-14-9	Ketones	-	0.25±0.03	0.35±0.14	0.43±0.04	0.38±0.08	1.06±0.11	1.69±0.28	0.61±0.13	0.71±0.10	1.14±0.36	0.74±0.16	8.32E-10	0.45
2-Undecenal	$C_{11}H_{20}O$	53448-07-0	Aldehydes	4.13±0.83	1.33±0.22	2.27±0.65	3.36±0.40	2.88±0.16	7.00±1.09	11.06±2.53	6.61±1.17	6.26±0.12	13.10±3.78	14.82±3.98	4.21E-08	1.31
2-Butyl-2-octenal	$C_{12}H_{22}O$	13019-16-4	Aldehydes	-	-	0.24±0.07	0.34±0.01	-	-	-	-	-	-	-	2.06E-15	0.34
(E)-4,5-Epoxy-*(E)*-2-decenal	$C_{10}H_{16}O_2$	134454-31-2	Aldehydes	0.30±0.07	-	-	-	-	1.21±0.30	-	1.00±0.11	0.95±0.37	1.10±0.39	2.80±0.53	1.77E-11	0.64
Tetradecane	$C_{14}H_{30}$	629-59-4	Alkanes	0.40±0.08	-	0.13±0.02	0.14±0.01	-	1.55±0.28	-	-	--	-	2.59±0.40	7.71E-16	0.75
Caryophyllene	$C_{15}H_{24}$	87-44-5	Alkenes	0.99±0.07	-	-	-	-	-	-	-	-	-	-	3.99E-24	0.30
Humulene	$C_{15}H_{24}$	6753-98-6	Alkenes	0.90±0.07	-	-	-	-	-	-	-	-	-	-	3.21E-23	0.28
Hexanophenone	$C_{12}H_{16}O$	942-92-7	Ketones	0.49±0.09	0.39±0.08	0.44±0.09	0.88±0.26	0.64±0.14	1.93±0.15	2.78±0.58	1.14±0.30	1.45±0.28	2.50±0.66	1.62±0.44	1.92E-08	0.63

β-Selinene	$C_{15}H_{24}$	17066-67-0	Alkenes	0.42±0.05	0.34±0.14	-	-	-	-	-	-	-	-	-	3.26E-11	0.34
α-Muurolene	$C_{15}H_{24}$	10208-80-7	Alkenes	-	-	0.53±0.14	0.52±0.02	0.36±0.07	0.89±0.23	1.50±0.19	0.65±0.08	0.38±0.33	1.59±0.25	0.82±0.20	4.38E-10	0.55
γ-Cadinene	$C_{15}H_{24}$	39029-41-9	Alkenes	0.81±0.18	-	-	-	-	-	-	--	-	-	-	1.52E-13	0.27
δ-Cadinene	$C_{15}H_{24}$	483-76-1	Alkenes	1.42±0.26	0.24±0.13	0.62±0.16	0.73±0.02	0.52±0.10	1.36±0.38	1.99±0.23	0.87±0.15	0.74±0.33	1.79±0.30	-	1.17E-09	0.62
α-Calacorene	$C_{15}H_{20}$	21391-99-1	Alkenes	0.23±0.07	0.15±0.03	0.15±0.03	0.18±0.02	0.14±0.02	0.35±0.10	0.49±0.07	0.22±0.03	0.16±0.15	0.47±0.07	0.30±0.06	3.61E-06	0.25
Hexadecane	$C_{16}H_{34}$	544-76-3	Alkanes	0.33±0.09	-	-	-	-	0.33±0.13	-	-	-	-	0.89±0.13	1.44E-13	0.40
α-Cadinol	$C_{15}H_{26}O$	481-34-5	Alcohols	0.42±0.06	-	-	-	-	-	-	-	-	-	-	9.26E-18	0.19
2-Pentadecanone	$C_{15}H_{30}O$	2345-28-0	Ketones	0.15±0.05	0.08±0.03	-	-	-	0.25±0.04	-	0.22±0.05	0.28±0.13	-	0.32±0.05	6.44E-09	0.28

Note: “-” indicates that it was not detected. The variable importance in projection (VIP) was used to evaluate the contribution of each variable in the OPLS-DA model.

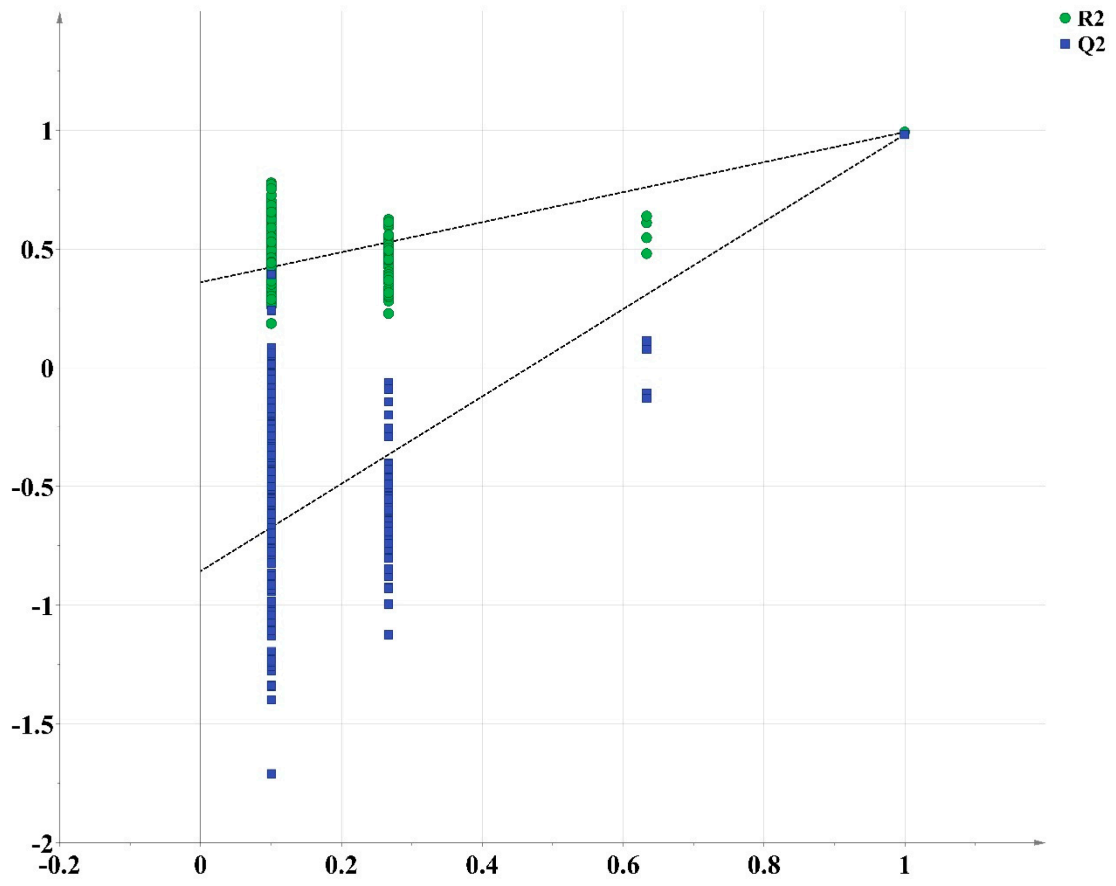

Figure S1. Results of 200 permutation tests

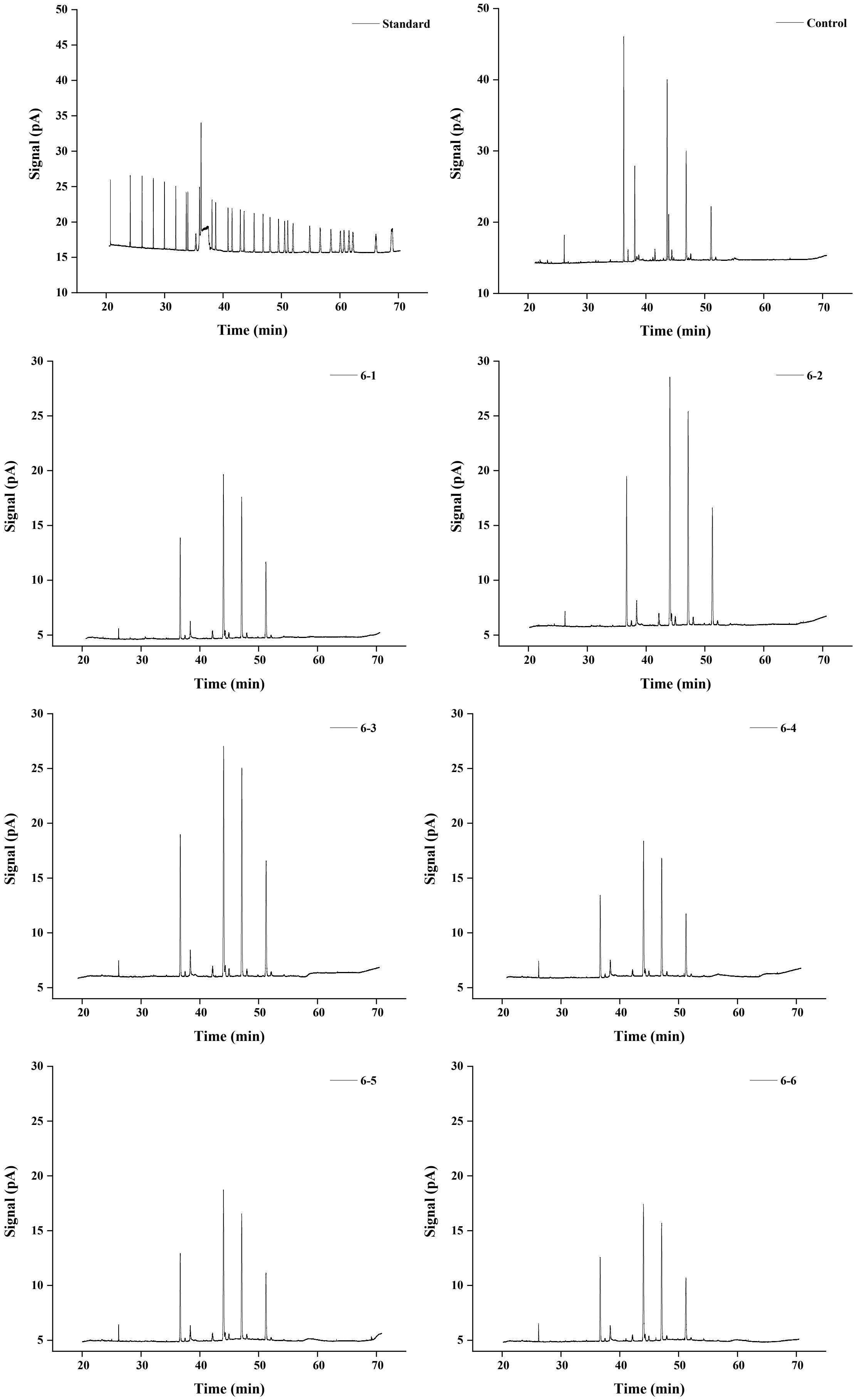
Standard
Control
6-1
6-2
6-3
6-4
6-5
6-6
Signal (pA)
Time (min)

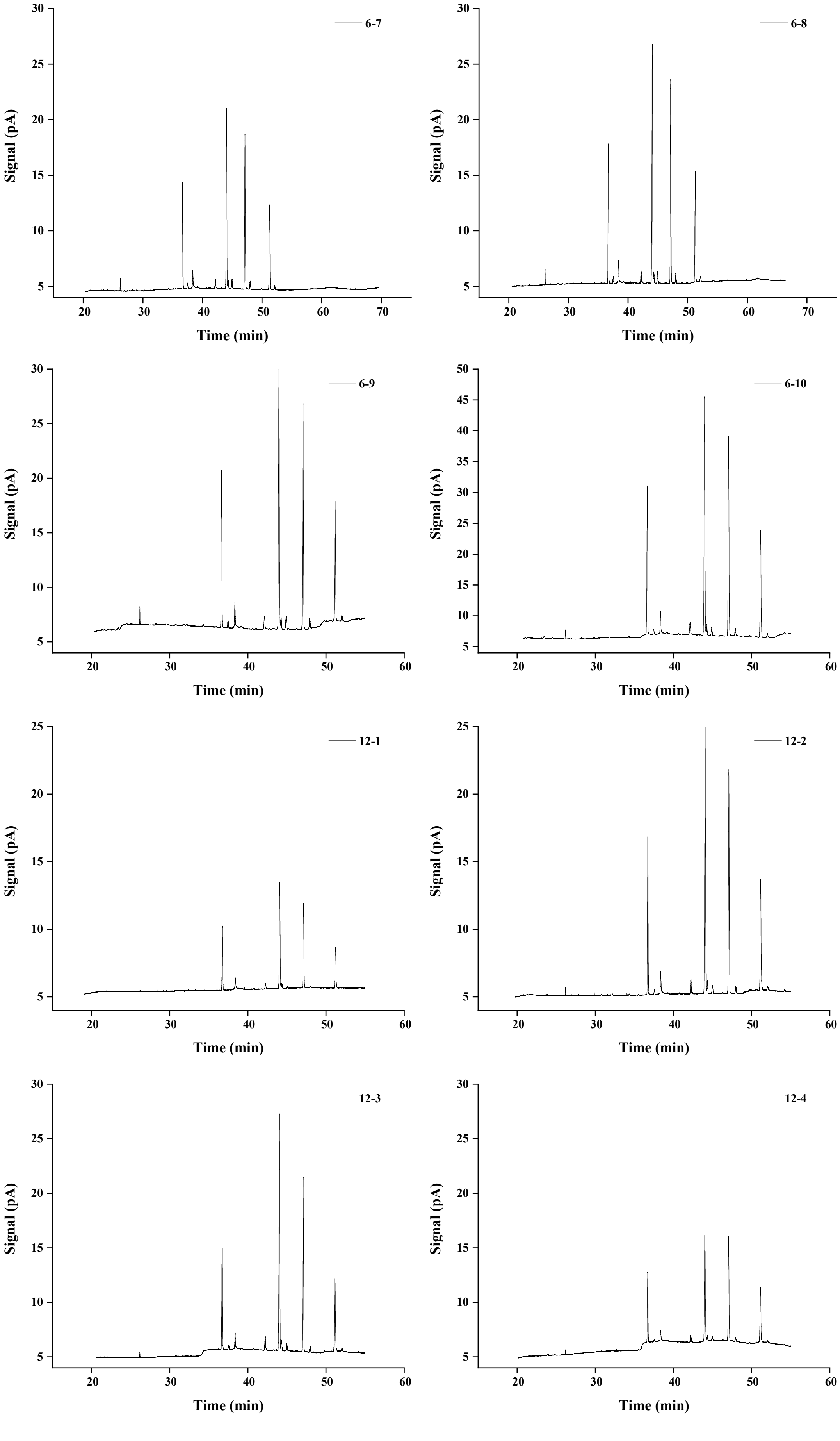
6-7
6-8
6-9
6-10
12-1
12-2
12-3
12-4
Signal (pA)
Time (min)

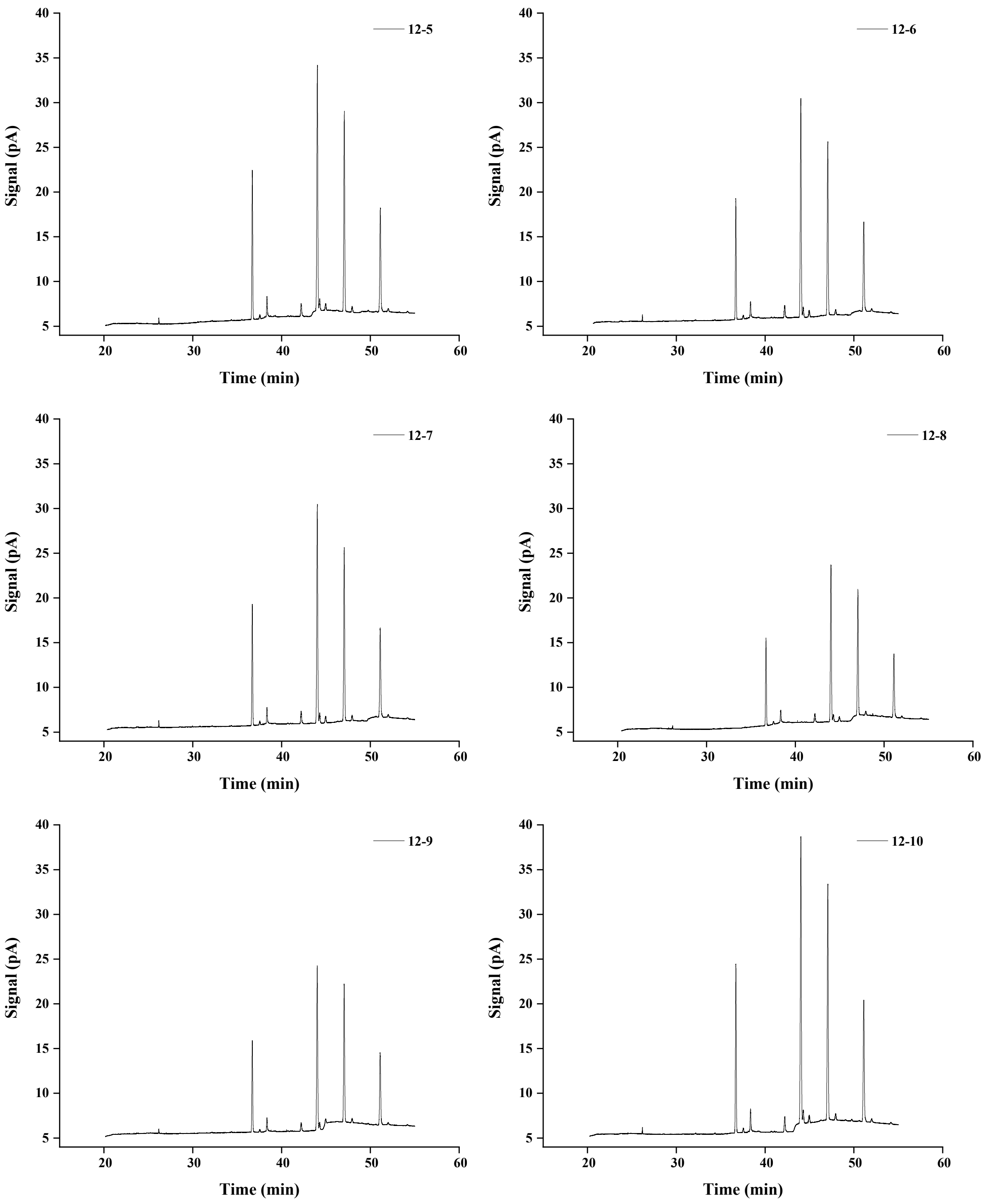

Figure S2. Chromatogram of fatty acids
